# Consistent Monocular Ackermann Visual–Inertial Odometry for Intelligent and Connected Vehicle Localization

**DOI:** 10.3390/s20205757

**Published:** 2020-10-10

**Authors:** Fangwu Ma, Jinzhu Shi, Liang Wu, Kai Dai, Shouren Zhong

**Affiliations:** State Key Laboratory of Automotive Simulation and Control, Jilin University, Changchun 130022, China; mikema@jlu.edu.cn (F.M.); shijz16@mails.jlu.edu.cn (J.S.); daikai18@mails.jlu.edu.cn (K.D.); zhongsr19@mails.jlu.edu.cn (S.Z.)

**Keywords:** visual–inertial odometry (VIO), lever arm effect, vehicle velocity and yaw angular rate error measurements, intelligent and connected vehicles

## Abstract

The observability of the scale direction in visual–inertial odometry (VIO) under degenerate motions of intelligent and connected vehicles can be improved by fusing Ackermann error state measurements. However, the relative kinematic error measurement model assumes that the vehicle velocity is constant between two consecutive camera states, which degrades the positioning accuracy. To address this problem, a consistent monocular Ackermann VIO, termed MAVIO, is proposed to combine the vehicle velocity and yaw angular rate error measurements, taking into account the lever arm effect between the vehicle and inertial measurement unit (IMU) coordinates with a tightly coupled filter-based mechanism. The lever arm effect is firstly introduced to improve the reliability for information exchange between the vehicle and IMU coordinates. Then, the process model and monocular visual measurement model are presented. Subsequently, the vehicle velocity and yaw angular rate error measurements are directly used to refine the estimator after visual observation. To obtain a global position for the vehicle, the raw Global Navigation Satellite System (GNSS) error measurement model, termed MAVIO-GNSS, is introduced to further improve the performance of MAVIO. The observability, consistency and positioning accuracy were comprehensively compared using real-world datasets. The experimental results demonstrated that MAVIO not only improved the observability of the VIO scale direction under the degenerate motions of ground vehicles, but also resolved the inconsistency problem of the relative kinematic error measurement model of the vehicle to further improve the positioning accuracy. Moreover, MAVIO-GNSS further improved the vehicle positioning accuracy under a long-distance driving state. The source code is publicly available for the benefit of the robotics community.

## 1. Introduction

Intelligent and connected vehicles (ICVs) are a popular research topic in intelligent transportation systems [1,2]. The Global Navigation Satellite System (GNSS) is one of the most mature global positioning technologies for vehicle navigation [3]. However, the GNSS signal is vulnerable under tunnels, trees and other obstacles [4]. Providing real-time and accurate vehicle pose information in GNSS-denied environments is a necessary prerequisite for ICV navigation [5,6,7]. Visual–inertial odometry (VIO), which integrates a visual sensor and inertial measurement unit (IMU), can provide pose information with six degrees of freedom (DOF). It is widely used in the pose estimation of mobile robots owing to the advantages of favorable robustness, small size and low cost. VIO mainly consists of filter-based methods, including the Multi-Sensor Fusion Approach (MSF) [8], the Robust Visual-Inertial Odometry (ROVIO) [9], the Multi-State Constraint Kalman Filter (MSCKF) [10], Stereo-MSCKF [11], S-MSCKF [12], the Robocentric Visual-Inertial Odometry (R-VIO) [13], Schmidt-MSCKF [14], the Lightweight Hybrid Visual-Inertial Odometry (LARVIO) [15,16] and optimization-based methods including the Open Keyframe-based Visual-Inertial SLAM (OKVIS) [17], ORB-SLAM-VI [18], VINS-Mono [19], PL-VIO [20], ICE-BA [21], VI-DSO [22], VINS-Fusion [23,24], Basalt [25] and ORB-SLAM3 [26]. A detailed review of the VIO methods can be found in the literature [27,28].

However, the standard VIO is subjected to additional unobservable directions under constant acceleration and straight-line driving states or approximate combinations of the above two driving states of ground vehicles, resulting in larger pose-estimation errors [29,30,31]. To address this problem, there are some studies of integrating VIO algorithms with the information from the vehicle proprioceptive sensors, including the drive motor encoder sensor and steering wheel angle sensor. Wu et al. [30] proposed fusing wheel speed encoder measurements into VIO based on the square-root inverse sliding window filter (SR-ISWF) [32], which significantly improved positioning accuracy under special motions of ground differential steering robots. Li et al. [33] proposed a factor-graph-based, gyro-aided localization system by exploiting the wheel odometry and gyro measurements, which achieved better accuracy than ORB-SLAM [34,35]. KO-Fusion [36] was proposed to fuse the Mecanum wheel motion constraint into RGB-D SLAM for ground robots, which improved the robustness of SLAM. Zheng and Liu [37] proposed an SE(2)-constrained pose parameterization optimization-based VIO for ground vehicles, which obtained better accuracy under sharp-turn motion. Dang et al. [38] proposed a tightly-coupled VIO to fuse wheel encoder measurements by considering wheel slippage, which achieved great improvements in positioning accuracy. Quan et al. [39] proposed a tightly coupled monocular simultaneous localization and mapping (SLAM) system, VOSLAM, by integrating visual, odometer and gyroscope measurements, which ensured the system’s accuracy. The VOSLAM improved the robustness of initialization and faulty information by a map initialization method and reliable odometer measurements. Liu et al. [40] proposed a tightly coupled optimization-based VIO to combine a wheel encoder, IMU and visual sensor, which improved the robustness of initialization and positioning accuracy. To improve the positioning accuracy before the first turning in [40], Liu et al. [41] proposed a bidirectional trajectory computation method, which achieved a more accurate real-time trajectory. Zhang et al. [42] proposed a wheel odometry with motion manifold representation for ground robot localization, which achieved accurate 6D pose estimation. Ye et al. [43] proposed a robust pose estimation method with multi-camera, odometer and gyroscope measurements in a tightly coupled optimization framework, which had obvious advantages in loop-closure detection. Gang et al. [44] proposed a tightly coupled SLAM method using wheel speed, IMU and monocular vision, which solved the problem of an unobservable scale and improved the positioning robustness. Zuo et al. [45] proposed a kinematics-constrained VIO and provided detailed observability analysis for a skid-steering mobile robot, and the experimental results showed that the kinematic parameters were observable under general motion and online kinematic parameter estimation can significantly improve positioning accuracy. VINS-Vehicle [46] was proposed to fuse two DOF vehicle dynamics models into VIO based on the sliding window optimization method, which significantly enhanced the robustness and accuracy compared with existing VIO methods. Lee et al. [47] proposed a visual-inertial-wheel odometry system, VIWO, by integrating the measurements of IMU, camera and wheel odometry preintegration, which provided theoretical guidance for the localization of ground differential steering vehicle.

By integrating the information from the vehicle proprioceptive sensors, the above tightly coupled VIO methods can improve the observability of the VIO scale direction and further enhance the vehicle positioning accuracy. However, apart from in [46], the above methods have not taken enough advantage of the measurements from the steering wheel angle sensor, which is a low-cost built-in sensor in ICVs. Moreover, the above methods except [30,47] adopt an optimization-based backend, which requires higher computational complexity. In view of the efficient performance, the Ackermann Multi-State Constraint Kalman Filter (ACK-MSCKF) [48] was proposed for integrating Ackermann error state measurements and S-MSCKF in a tightly coupled filter-based mechanism in our previous work, which improved the positioning accuracy under degenerate motions of ground vehicles. However, it adopts a stereo configuration, which is more costly and less computationally efficient than monocular solutions. Moreover, it assumes that the vehicle velocity is constant between two consecutive camera states, which degrades the positioning accuracy. Lastly and most importantly, the observability, consistency and positioning accuracy of different parameter configurations of ACK-MSCKF with vehicle relative kinematic error measurements need to be further explored.

Therefore, in this paper, a tightly coupled filter-based consistent monocular Ackermann VIO, termed MAVIO, is proposed to combine the vehicle velocity and yaw angular rate error measurements, considering the lever arm effect between the vehicle and IMU coordinates. Similar to in the work [49], to obtain a global position for the vehicle, the raw GNSS error measurement model, termed MAVIO-GNSS, is introduced to further improve the performance of MAVIO. The famous open-source VIO, i.e., S-MSCKF [12], is adopted as the base of this work. The main contributions of this paper are highlighted as follows:

(1) Additional analyses of different parameter configurations of ACK-MSCKF are performed with more real-world experiments.
(2) Conducting the formulation and implementation of a consistent monocular Ackermann VIO, MAVIO, which not only improves the observability of the VIO scale direction but also resolves the inconsistency problem of ACK-MSCKF for further improving the positioning accuracy.
(3) Introducing the raw GNSS error measurement model, MAVIO-GNSS, which further improves the vehicle positioning accuracy under the long-distance driving state.
(4) The performance of MAVIO and MAVIO-GNSS are comprehensively compared with S-MSCKF and ACK-MSCKF on real-world datasets with twenty rounds, on average, of real-world experiments.
(5) The source code [50] of MAVIO is publicly available to facilitate the reproducibility of related research.

The remainder of this paper is structured as follows: The proposed approach is introduced in detail in Section 2. Then, Section 3 describes how the real-world experiments were carried out. Besides, the experimental results are discussed in Section 4. Finally, Section 5 presents the conclusions drawn.

## 2. The Proposed Approach

### 2.1. Coordinate Systems and Notations

The Vehicle Coordinate System {B}, IMU Coordinate System {I} and Camera Coordinate System {C} had the same definitions as in [48]. The additional four coordinate systems were defined as follows:

(1) Inertial Coordinate System of IMU {G_I_}. The origin of {G_I_} is the same as that of {I} at the time of VIO initialization. The axes of {G_I_} are obtained by calculation at the time of VIO initialization, and its *z*-axis is aligned with Earth’s gravity.
(2) Inertial Coordinate System of Vehicle {G_B_}. The origin of {G_B_} is the same as that of {B} at the time of VIO initialization. The axes of {G_B_} are obtained by calculation at the time of VIO initialization, and its *z*-axis is aligned with Earth’s gravity.
(3) GNSS Coordinate System {S}. The origin of {S} lies in the center of the GNSS equipment. The *x*-axis and *y*-axis point forward and to the right, respectively, following the right-hand rule.
(4) Universal Transverse Mercator Coordinate System {U_S_}. The {U_S_} is a universal global coordinate system. Please refer to [51] for more details.

In this paper, we adopted the Jet Propulsion Laboratory (JPL) Proposed Standard Conventions [52,53] to derive the corresponding formulas.

### 2.2. The Lever Arm Effect between the Vehicle and IMU Coordinates

**Figure 1:**
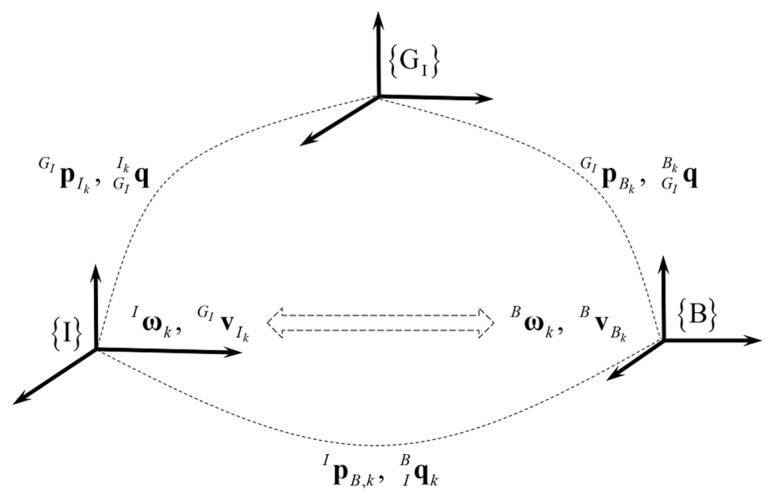
The lever arm effect between {B} and {I}. qGIIk denotes the rotation quaternion from {G_I_} to {I} at time $t_{k}$; pGIIk denotes the position of {I} in {G_I_} at time $t_{k}$. qGIBk denotes the rotation quaternion from {G_I_} to {B} at time $t_{k}$; pGIBk denotes the position of {B} in {G_I_} at time $t_{k}$. qIBk and pIB,k denote the rotation and translation extrinsic parameters between {B} and {I} at time $t_{k}$, respectively. vGIIk denotes the velocity of {I} in {G_I_} at time $t_{k}$; vBBk denotes the vehicle velocity at time $t_{k}$. ωIk and ωBk denote the angular rates of {I} and {B} at time $t_{k}$, respectively.

In the practical application of VIO systems for vehicles, the IMU and camera are usually installed on the front of the vehicle. Due to the existence of translation extrinsic parameters between {B} and {I}, the lever arm effect between {B} and {I} cannot be negligible. The lever arm effect between {B} and {I} is shown in Figure 1.

According to the presentation of the lever arm effect in [54], the vehicle velocity vBBk at time $t_{k}$ is derived as

(1) vBBk=C(qGIBk)vGIIk+vBlk

where vBlk denotes the lever arm velocity at time $t_{k}$ between {B} and {I}, vBlk and C(qGIBk) are defined as

(2) {vBlk=C(qIBk)(p˙IB,k+⌊ωIk×⌋pIB,k)C(qGIBk)=C(qIBk)⋅C(qGIIk)

and the vehicle angular ωBk rate at time $t_{k}$ is derived as

(3) ωBk=C(qIBk)⋅ωIk+ωIib,k

where ωIib,k denotes the relative angular rate between {B} and {I}, which can be negligible with a fixed installed location for the IMU.

### 2.3. Process Model and Monocular Visual Measurement Model

The vehicle state vector $X_{B_{k}}$ at the sampling time for the IMU $t_{k}$ is defined as [54]

(4) XBk=[qGBBkTpGBBkTvGBIkTqIBkTpIB,kTbg,kTba,kT]T

where qGBBk denotes the rotation quaternion from {G_B_} to {B} at time $t_{k}$. pGBBk denotes the position of {B} in {G_B_}. vGBIk denotes the velocity of {I} in {G_B_}. qIBk and pIB,k denote the rotation and translation extrinsic parameters between {B} and {I} at time $t_{k}$, respectively. The symbols $b_{g,k}^{}$ and $b_{a,k}^{}$ denote the gyroscope and accelerometer biases of the IMU at time $t_{k}$, respectively.

Following Equation (4), the error state vector ${\tilde{X}}_{B_{k}}\in {\mathbb{R}}^{21}$ at time $t_{k}$ is defined as

(5) X˜Bk=[θ˜GBBkTp˜GBBkTv˜GBIkTθ˜IBkTp˜IB,kTb˜g,kTb˜a,kT]T

The continuous-time kinematic differential equations of the true state are

(6) {q˙GBBk=12Ω(C(qIBk)ωIk)qGBBkp˙GBBk=vGBIk+C(qGBBk)TC(qIBk)(⌊ωIk×⌋pIB,k)v˙GBIk=C(qGBBk)TC(qIBk)aIk+gGq˙IBk=03×1, p˙IB,k=03×1b˙g,k=nωg,k b˙a,k=nωa,k

where $n_{\omega g,k}$ and $n_{\omega a,k}^{}$ denote the random-walk noises of the gyroscope and accelerometer biases at time $t_{k}$, respectively. The true IMU angular rate ωIk and acceleration aIk at time $t_{k}$ are represented as

(7) {ωIk=ωIm,k−bg,k−ng,kaIk=aIm,k−ba,k−na,k

where $n_{g,k}$ and $n_{a,k}$ denote the Gaussian white noises of the gyroscope and accelerometer measurements at time $t_{k}$, respectively.

According to Equation (6), the linearized continuous-time kinematic differential equation of the error state at time $t_{k}$ follows

(8) $${\dot{\tilde{X}}}_{B_{k}}=F^{B_{k}}{\tilde{X}}_{B_{k}}+G^{B_{k}}n^{B_{k}}$$

where $n_{B_{k}}={\left[\begin{array}{llll} n_{g,k}^{T} & n_{\omega g,k}^{T} & n_{a,k}^{T} & n_{\omega a,k}^{T} \end{array}\right]}^{T}$ denotes the continuous-time input noise vector. $F^{B_{k}}$ and $G^{B_{k}}$ are the continuous-time error-state transition matrix and input noise Jacobian matrix at time $t_{k}$, respectively. $F^{B_{k}}$ and $G^{B_{k}}$ are represented as

(9) $$F^{B_{k}}=\left[\begin{array}{ccccccc} F_{\left(1,1\right)}^{B_{k}} & 0_{3\times 3} & 0_{3\times 3} & F_{\left(1,4\right)}^{B_{k}} & 0_{3\times 3} & F_{\left(1,6\right)}^{B_{k}} & 0_{3\times 3}\\ F_{\left(2,1\right)}^{B_{k}} & 0_{3\times 3} & F_{\left(2,3\right)}^{B_{k}} & F_{\left(2,4\right)}^{B_{k}} & F_{\left(2,5\right)}^{B_{k}} & F_{\left(2,6\right)}^{B_{k}} & 0_{3\times 3}\\ F_{\left(3,1\right)}^{B_{k}} & 0_{3\times 3} & 0_{3\times 3} & F_{\left(3,4\right)}^{B_{k}} & 0_{3\times 3} & 0_{3\times 3} & F_{\left(3,7\right)}^{B_{k}}\\ 0_{3\times 3} & 0_{3\times 3} & 0_{3\times 3} & 0_{3\times 3} & 0_{3\times 3} & 0_{3\times 3} & 0_{3\times 3}\\ 0_{3\times 3} & 0_{3\times 3} & 0_{3\times 3} & 0_{3\times 3} & 0_{3\times 3} & 0_{3\times 3} & 0_{3\times 3}\\ 0_{3\times 3} & 0_{3\times 3} & 0_{3\times 3} & 0_{3\times 3} & 0_{3\times 3} & 0_{3\times 3} & 0_{3\times 3}\\ 0_{3\times 3} & 0_{3\times 3} & 0_{3\times 3} & 0_{3\times 3} & 0_{3\times 3} & 0_{3\times 3} & 0_{3\times 3} \end{array}\right]$$

(10) $$G^{B_{k}}=\left[\begin{array}{cccc} G_{\left(1,1\right)}^{B_{k}} & 0_{3\times 3} & 0_{3\times 3} & 0_{3\times 3}\\ G_{\left(2,1\right)}^{B_{k}} & 0_{3\times 3} & 0_{3\times 3} & 0_{3\times 3}\\ 0_{3\times 3} & 0_{3\times 3} & G_{\left(3,3\right)}^{B_{k}} & 0_{3\times 3}\\ 0_{3\times 3} & 0_{3\times 3} & 0_{3\times 3} & 0_{3\times 3}\\ 0_{3\times 3} & 0_{3\times 3} & 0_{3\times 3} & 0_{3\times 3}\\ 0_{3\times 3} & G_{\left(6,2\right)}^{B_{k}} & 0_{3\times 3} & 0_{3\times 3}\\ 0_{3\times 3} & 0_{3\times 3} & 0_{3\times 3} & G_{\left(7,4\right)}^{B_{k}} \end{array}\right]$$

where the analytical expressions of each block matrix in $F^{B_{k}}$ and $G^{B_{k}}$ are derived as described in Appendix A.

The camera state at the sampling time of camera $t_{j}$ is defined as

(11) XCj=[qGBCjTpGBCjT]T

where qGBCj denotes the rotation quaternion from {G_B_} to {C} at time $t_{j}$ and pGBCj is the position of {C} in {G_B_}. At $t_{k}=t_{j}$, qGBCj and pGBCj are given by

(12) {qGBCj=qIC⊗qBIk⊗qGBBkpGBCj=pGBBk+C(qGBBk)TC(qIBk)(pIC−pIB,k)

According to Equation (4) and Equation (11), the full state vector $X_{k}$ with N camera states at time $t_{k}\in \left[\begin{array}{cc} t_{j} & t_{j+1} \end{array}\right)$ is given by

(13) Xk=[XBkTqGBCj−N+1TpGBCj−N+1TqGBCj−N+2TpGBCj−N+2T⋯qGBCj−1TpGBCj−1TqGBCjTpGBCjT]T

Following Equation (13), the full error state vector ${\tilde{X}}_{k}\in {\mathbb{R}}^{21+6N}$ at time $t_{k}$ is defined as

(14) X˜k=[X˜BkTθ˜GBCj−N+1Tp˜GBCj−N+1Tθ˜GBCj−N+2Tp˜GBCj−N+2T⋯θ˜GBCj−1Tp˜GBCj−1Tθ˜GBCjTp˜GBCjT]T

The augmented state covariance matrix and monocular visual feature measurement model can be obtained as detailed in [10,12].

### 2.4. Kinematic Error Measurement Model for Vehicle

#### 2.4.1. Measurements of Vehicle Relative Kinematic Error

The refined measurement Jacobian matrix $H_{}^{B_{j}}$ of the vehicle relative kinematic errors in Equation (18) of [48] is represented as

(15) $$H_{}^{B_{j}}=\left[\begin{array}{cccccccc} 0_{3\times 15} & H_{\left(1,2\right)}^{B_{j,\theta }} & 0_{3\times 3} & 0_{3\times \left(6N-12\right)} & H_{\left(1,5\right)}^{B_{j,\theta }} & 0_{3\times 3} & H_{\left(1,7\right)}^{B_{j,\theta }} & 0_{3\times 3}\\ 0_{3\times 15} & H_{\left(2,2\right)}^{B_{j,v}} & H_{\left(2,3\right)}^{B_{j,v}} & 0_{3\times \left(6N-12\right)} & H_{\left(2,5\right)}^{B_{j,v}} & H_{\left(2,6\right)}^{B_{j,v}} & H_{\left(2,7\right)}^{B_{j,v}} & H_{\left(2,8\right)}^{B_{j,v}} \end{array}\right]$$

where $H_{\left(1,2\right)}^{B_{j,\theta }},\;H_{\left(1,5\right)}^{B_{j,\theta }}\;\mathrm{and}\;H_{\left(1,7\right)}^{B_{j,\theta }}$ denote the measurement Jacobian block matrixes of the vehicle relative rotation errors; $H_{\left(2,2\right)}^{B_{j,v}},\;H_{\left(2,3\right)}^{B_{j,v}},\;H_{\left(2,5\right)}^{B_{j,v}},\;H_{\left(2,6\right)}^{B_{j,v}},\;H_{\left(2,7\right)}^{B_{j,v}}\;\mathrm{and}\;H_{\left(2,8\right)}^{B_{j,v}}$ denote the measurement Jacobian block matrixes of the vehicle relative translation errors; and their analytical expressions are derived as described in Appendix B.

Considering the fact that the measurements of vehicle kinematic error originate from the vehicle velocity, angular rate, and the characteristics of the vehicle relative kinematic errors between two consecutive camera states, the method using a kinematic error measurement model for the vehicle has different parameter configurations in practical application. Therefore, following Equation (15), the tunable parameter configurations of ACK-MSCKF are as shown in Table 1.

#### 2.4.2. Measurements of Vehicle Velocity and Angular Rate Error

The limitation of the above relative kinematic error measurement model for the vehicle derives from the assumption that the vehicle velocity is constant for low-speed motion between two consecutive camera states. To address this problem, the vehicle velocity and yaw angular rate error measurement model, taking into account the lever arm effect between {B} and {I}, is presented as one of the primary contributions of this paper.

The measurement residual $r^{B_{k}}$ at time $t_{k}$ is defined as

(16) rBk=(rBωkrBvk)=(ωBk−ω^BkvBBk−v^BBk)

where rBvk and rBωk denote the vehicle velocity measurement residual and angular rate measurement residual at time $t_{k}$ in {B}, respectively. vBBk and ωBk denote the vehicle velocity and angular rate measurements at time $t_{k}$ in {B}, respectively. v^BBk and ω^Bk denote the vehicle velocity and angular rate estimations at time $t_{k}$ in {B}, respectively.

Considering the nonholonomic constraint of ground vehicles [54], vBBk and ωBk are represented as

(17) {vBBk=[vBBk,x00]TωBk=[00ωBk,z]T

where vBBk,x and ωBk,z denote the vehicle’s longitudinal speed and the yaw angular rate measurements at time $t_{k}$ in {B}, respectively. vBBk,x can be obtained from the vehicle controller area network-bus (CAN-bus), and ωBk,z is represented as

(18) ωBk,z=vBBk,x/Rk

where $R_{k}$ denotes the steering radius at time $t_{k}$; it can be obtained according to the Ackermann steering geometry [55]:

(19) $$R_{k}=\frac{2L-B\tan\alpha_{o,k}}{2\tan\alpha_{o,k}}$$

Following Equation (19), ωBk,z is represented as

(20) ωBk,z=vBBk,x/Rk=2vBBk,xtanαo,k2L−Btanαo,k

where $R_{k}$ denotes the steering radius at time $t_{k}$. L and B denote the wheel base and the distance between the steering king pins, respectively, which can be acquired from manual measurement. $\alpha_{o,k}$ denotes the outer front wheel steering angle at time $t_{k}$, which is represented as

(21) $$\alpha_{o,k}=\delta_{o,k}/\lambda$$

where λ denotes the steering angular transmission ratio obtained from the offline calibration. $\delta_{o,k}$ denotes the steering wheel angle at time $t_{k}$, which can also be obtained from the vehicle CAN-bus.

Based on the lever arm effect between {B} and {I}, ω^Bk and v^BBk are represented as

(22) {ω^Bk=C(q^IBk)⋅ω^Ikv^BBk=C(q^GBBk)v^GBIk+C(q^IBk)(⌊ω^Ik×⌋p^IB,k)

By linearizing Equation (16) at current error state ${\tilde{X}}_{k}$, $r_{B_{k}}$ is approximated as

(23) $$r^{B_{k}}≃H^{B_{k}}{\tilde{X}}_{k}+n^{B_{k}}$$

where $n^{B_{k}}$ denotes the measurement noise vector at time $t_{k}$:

(24) $$n^{B_{k}}={\left[\begin{array}{cccccc} n_{\omega x}^{B} & n_{\omega y}^{B} & n_{\omega z}^{B_{k}} & n_{vx}^{B} & n_{vy}^{B} & n_{vz}^{B} \end{array}\right]}^{T}$$

where $n_{\omega x}^{B},\;n_{\omega y}^{B}\;\mathrm{and}\;n_{\omega z}^{B_{k}}$ denote the measurement noises of ωBk, and $n_{vx}^{B},\;n_{vy}^{B}\;\mathrm{and}\;n_{vz}^{B}$ denote the measurement noises of vBBk. The measurement covariance matrix of $n^{B_{k}}$ is given by

(25) $$U_{k}=diag\left\{\begin{array}{cccccc} \sigma_{B,\omega x}^{2} & \sigma_{B,\omega y}^{2} & U_{\omega z}^{B_{k}} & \sigma_{B,vx}^{2} & \sigma_{B,vy}^{2} & \sigma_{B,vz}^{2} \end{array}\right\}$$

where $\sigma_{B,vx}^{},\;\sigma_{B,vy}^{},\;\sigma_{B,vz}^{},\;\sigma_{B,\omega x}^{}\;\mathrm{and}\;\sigma_{B,\omega y}^{}$ denote the standard deviations of the measurement noise. $U_{\omega z}^{B_{k}}$ denotes the variance of $n_{\omega z}^{B_{k}}$ at time $t_{k}$; it is obtained by

(26) $$U_{\omega z}^{B_{k}}=Z_{k}VZ_{k}^{T}+Q_{\omega z}^{B_{k}}$$

where $Q_{\omega z}^{B_{k}}$ denotes the variance of additional uncertain noise, and V denotes the covariance matrix of the input noise:

(27) $$V=\left[\begin{array}{cc} \sigma_{\alpha }^{2} & 0\\ 0 & \sigma_{B,vx}^{2} \end{array}\right]$$

where $\sigma_{\alpha }$ denotes the standard deviation of the outer front wheel steering angle.

$Z_{k}$ in Equation (26) denotes the Jacobian matrix of the input noise; it is represented as

(28) $$Z_{k}=\left[\begin{array}{cc} Z_{k,\alpha } & Z_{k,vx} \end{array}\right]$$

where

(29) {Zk,α=∂ωBk,z∂αo,k=LvBBk,x(tan2αo,k+1)(Rktanαo,k)2Zk,vx=∂ωBk,z∂vBBk,x=1Rk

$H^{B_{k}}$ in Equation (23) represents the measurement Jacobian matrix of vehicle velocity and angular rate errors; it is derived as

(30) $$H^{B_{k}}=\left[\begin{array}{cccccccc} 0_{3\times 3} & 0_{3\times 3} & 0_{3\times 3} & H_{\left(1,4\right)}^{B_{k},\omega } & 0_{3\times 3} & H_{\left(1,6\right)}^{B_{k},\omega } & 0_{3\times 3} & 0_{3\times 6N}\\ H_{\left(2,1\right)}^{B_{k},v} & 0_{3\times 3} & H_{\left(2,3\right)}^{B_{k},v} & H_{\left(2,4\right)}^{B_{k},v} & H_{\left(2,5\right)}^{B_{k},v} & H_{\left(2,6\right)}^{B_{k},v} & 0_{3\times 3} & 0_{3\times 6N} \end{array}\right]$$

where $H_{\left(1,4\right)}^{B_{k},\omega }\;\mathrm{and}\;H_{\left(1,6\right)}^{B_{k},\omega }$ denote the measurement Jacobian block matrixes of the vehicle angular rate errors; $H_{\left(2,1\right)}^{B_{k},v},\;H_{\left(2,3\right)}^{B_{k},v},\;H_{\left(2,4\right)}^{B_{k},v},\;H_{\left(2,5\right)}^{B_{k},v}\;\mathrm{and}\;H_{\left(2,6\right)}^{B_{k},v}$ denote the measurement Jacobian block matrixes of the vehicle velocity errors; and their analytical expressions are derived as described in Appendix C.

Following Equation (30), the tunable parameter configurations of MAVIO are shown in Table 2.

Following Equations (8) and (23), the updated covariance matrix and state vector of MAVIO at time $t_{k}$ are obtained by using the standard extended Kalman filter (EKF).

### 2.5. Raw GNSS Error Measurement Model

To obtain a global position for the vehicle, the raw GNSS error measurement model is proposed to further refine the estimator after the process of MAVIO(1).

The measurement residual rUSpk at time $t_{k}$ is defined as

(31) rUSpk=pUSSk−p^USSk

where pUSSk and p^USSk denote the measured and estimated values of {S} in {U_S_} at time $t_{k}$, respectively. pUSSk can be converted from the raw latitude and longitude of the dual-antenna Spatial NAV982-RG inertial navigation system without the carrier-phase based differential process by using the robot_localization [56] process. p^USSk is derived as

(32) p^USSk=pUSGB+C(qUSGB)Tp^GBSk=pUSGB+C(qUSGB)T(p^GBBk+C(q^GBBk)Tp^BS,k)=pUSGB+C(qUSGB)Tp^GBBk+C(qUSGB)TC(q^GBBk)TC(q^IBk)(pIS−p^IB,k)

where qUSGB denotes the rotation quaternion from {U_S_} to {G_B_}. pUSGB denotes the position of {G_B_} in {U_S_}. pIS denotes the translation extrinsic parameter between {S} and {I}.

By linearizing Equation (32) at current error state ${\tilde{X}}_{k}$, rUSpk is approximated as

(33) rUSpk≃HSkX˜k+nSk

where $n^{S_{k}}$ denotes the measurement noise vector of the raw GNSS data at time $t_{k}$. $H^{S_{k}}$ represents the measurement Jacobian matrix of the raw GNSS errors; it is derived as

(34) $$H^{S_{k}}=\left[\begin{array}{cccccccc} H_{\left(1,1\right)}^{S_{k},p} & H_{\left(1,2\right)}^{S_{k},p} & 0_{3\times 3} & H_{\left(1,4\right)}^{S_{k},p} & H_{\left(1,5\right)}^{S_{k},p} & 0_{3\times 3} & 0_{3\times 3} & 0_{3\times 6N} \end{array}\right]$$

where $H_{\left(1,1\right)}^{S_{k},p},\;H_{\left(1,2\right)}^{S_{k},p},\;H_{\left(1,4\right)}^{S_{k},p}\;\mathrm{and}\;H_{\left(1,5\right)}^{S_{k},p}$ denote the measurement Jacobian block matrixes of the raw GNSS errors, and their analytical expressions are derived as described in Appendix D.

Following Equations (8) and (33), the updated covariance matrix and state vector of MAVIO-GNSS at time $t_{k}$ are obtained by using the standard extended Kalman filter (EKF).

## 3. Experiments and Results

### 3.1. Experimental Vehicle Platform and Real-World Datasets

**Figure 2:**
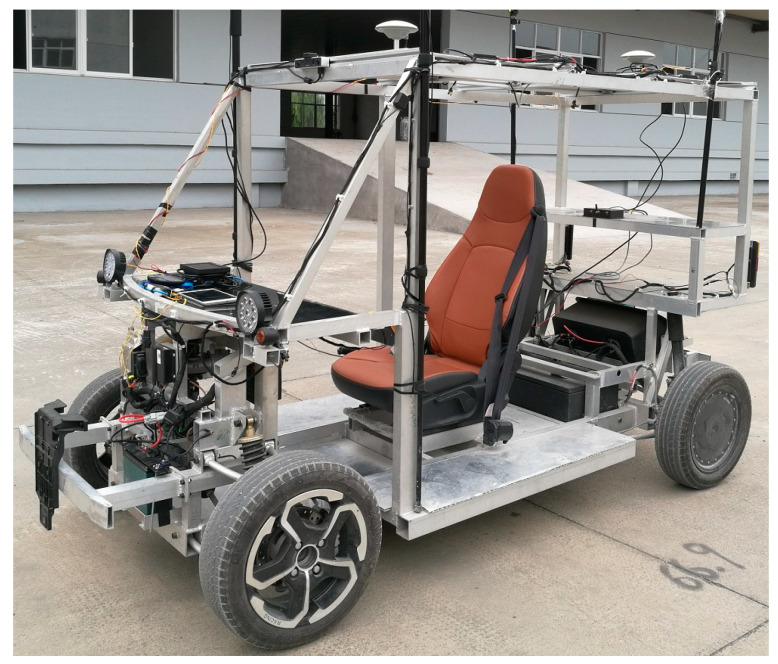
Experimental vehicle platform: Vehicle_a27.

The real-world experiments were performed with a medium-size Ackermann steering vehicle, i.e., Vehicle_a27, which is shown in Figure 2. For further details of the experimental vehicle setup, please refer to [48].

Taking into account the different vehicle driving states and travel distances, six different real-world experimental datasets were acquired by using Vehicle_a27, i.e., the VD01 dataset, VD02 dataset, VD03 dataset, VD04 dataset, VD05 dataset and VD06 dataset. The VD01 dataset and VD02 dataset were, respectively, acquired under a straight-line driving state and an S-shaped-curve driving state. The VD03 dataset was acquired under a circular-curve driving state for five circles. The VD04 dataset was acquired under straight-line and turning driving states by traveling around the building in a test field for one circle. The VD05 dataset was acquired under S-shaped-curve, straight-line and turning driving states by traveling around the building in a test field for one circle. The VD04 and VD05 datasets correspond to the AM_01 and AM_02 datasets in [48], respectively. The VD06 dataset was acquired under straight-line and turning driving states by traveling around the building in a test field for three circles. The details of the real-world experimental datasets are shown in Table 3.

**Figure 3:**
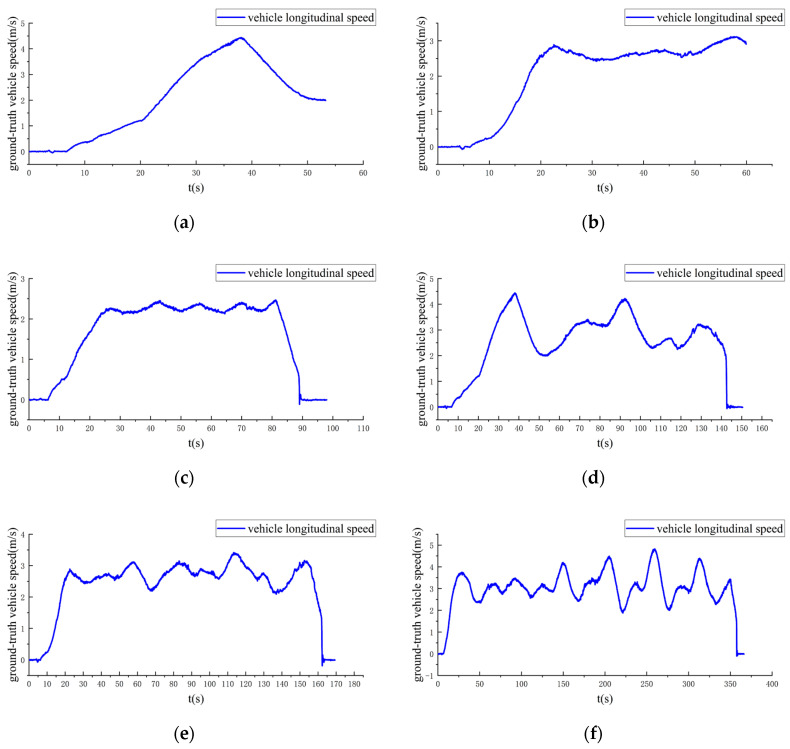
The ground-truth vehicle longitudinal speeds for the datasets: (**a**) VD01; (**b**) VD02; (**c**) VD03; (**d**) VD04; (**e**) VD05; (**f**) VD06.

Figure 3 shows the ground-truth vehicle longitudinal speeds for the real-world datasets.

Figure 3 indicates that the ground-truth vehicle longitudinal acceleration was close to a constant value in short periods of time for all of the real-world datasets, which provides potential support for the data with degenerate motions of ground vehicles.

### 3.2. Experimental Results

Considering the differences between each round of real-world experimental results caused by the non-real-time computer operating system and other uncertain factors, the performance of MAVIO and MAVIO-GNSS were compared with S-MSCKF and ACK-MSCKF using real-world datasets by averaging all twenty rounds of experimental results. The arithmetic mean was adopted for the averaging of many rounds of vehicle position and root mean square scale ratio data. For the average of the vehicle attitude data, the weighted average quaternion [57] was used. For qualitative and quantitative positioning accuracy evaluation, the estimated trajectory, absolute trajectory error (ATE), relative translation error (RTE) and relative translation error percentage (RTEP) were calculated based on the average results by using the rpg_trajectory_evaluation [58] package. Owing to this operation, the results in this paper are more convincing. The source code for the data average processing can be publicly accessed from [59].

For consistency of comparison, we adopted the ground-truth pose errors and estimated ±3σ (triple standard deviation) bounds [60] along with time in one round of experiments. For observability comparisons for scale direction, the root mean square scale ratio (RMSSR) $S_{rmssr,k}^{}$ at time $t_{k}$ was used as an evaluation index. The smaller the root mean square scale ratio $S_{rmssr,k}^{}$, the more consistent the estimated scale of the positioning trajectory with the real scale [54]. $S_{rmssr,k}^{}$ is defined as

(35) $$S_{rmssr,k}^{}=\sqrt{\frac{1}{k-1}\sum_{i=2}^{k}S_{i}^{2}}$$

where k denotes the number of output pose estimation results. $S_{i}$ denotes the scale ratio [30] at time $t_{i}$

(36) $$S_{i}=\{\begin{array}{ll} \frac{\Delta {\hat{d}}_{i}}{\Delta d_{i}}-1\; & \mathrm{for}\;\Delta {\hat{d}}_{i}>\Delta d_{i}\\ -\left(\frac{\Delta d_{i}}{\Delta {\hat{d}}_{i}}-1\right) & \mathrm{for}\Delta d_{i}>\Delta {\hat{d}}_{i} \end{array}$$

where,

(37) Δd^i=p^Bi−1BiT⋅p^Bi−1Bi=(p^GBBi−p^GBBi−1)T⋅(p^GBBi−p^GBBi−1)Δdi=pBi−1BiT⋅pBi−1Bi=(pGBBi−pGBBi−1)T⋅(pGBBi−pGBBi−1)

where p^GBBi and p^GBBi−1 denote the estimated positions of {B} in {G_B_} at time $t_{i}$ and $t_{i-1}$, respectively. pGBBi and pGBBi−1 denote the ground-truth positions of {B} in {G_B_} at time $t_{i}$ and $t_{i-1}$, respectively.

qUSGB and pUSGB are initialized by the trajectory alignment method [61]; the open source code [62] is referenced in the implementation of the trajectory alignment. For simplicity, the origin of {B} coincides with that of {S}. The initial translation extrinsic parameter vector pIB between {B} and {I} is obtained by manual measurement. The initial rotation extrinsic parameter vector qIB between {B} and {I} was calibrated by the method proposed in Section 2.4 of [48]. Taking into account the effects of vehicle vibration and temperature changes, the IMU noise parameters calibrated with the kalibr_allan [63] package were expanded by 10 times in all the real-world experiments.

#### 3.2.1. Observability and Consistency Comparison

**Figure 4:**
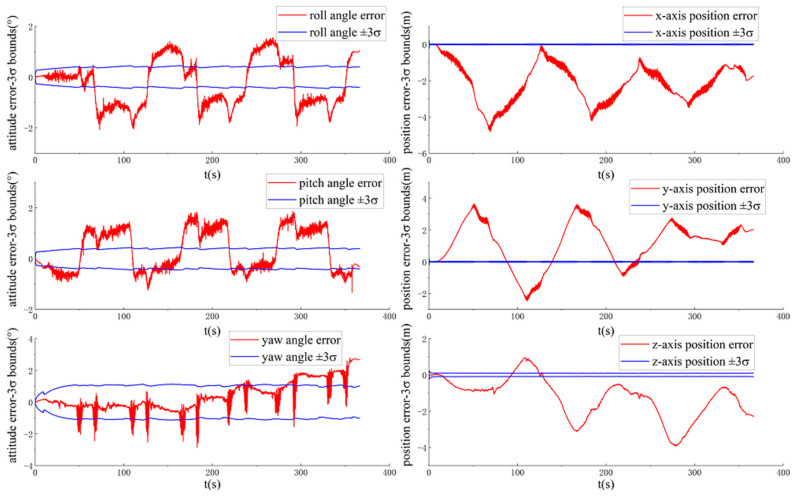
The ground-truth pose errors and estimated ±3σ bounds of ACK-MSCKF(1) with time for the VD06 dataset.

**Figure 5:**
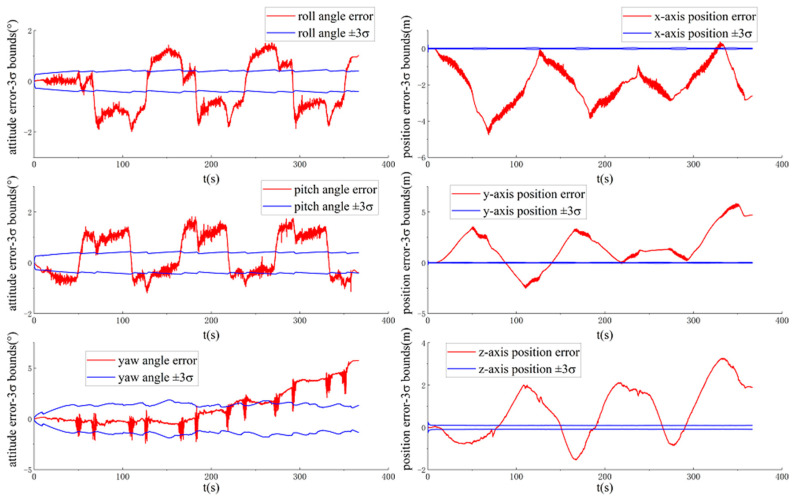
The ground-truth pose errors and estimated ±3σ bounds of ACK-MSCKF(2) with time for the VD06 dataset.

Figure 4 and Figure 5 show the ground-truth pose errors and estimated ±3σ bounds of ACK-MSCKF(1) and ACK-MSCKF(2) with time, respectively.

Figure 4 and Figure 5 show that the estimated three-axis position standard deviation of ACK-MSCKF(1) and ACK-MSCKF(2) with time for the VD06 dataset is close to zero, and the estimated three-axis attitude angle standard deviation tends to be constant with time. These results indicate that the three-axis vehicle position error state p˜GBB and attitude angle error state θ˜GBB are both observable. Actually, the vehicle relative kinematic error measurements do not provide information on the vehicle yaw angle and three-axis position in {G_B_}. Therefore, ACK-MSCKF(1) and ACK-MSCKF(2) obtain spurious information along the direction of the vehicle yaw angle and three-axis position, which leads to an inconsistency problem in that the ground-truth errors of the vehicle yaw angle and three-axis position are far greater than their estimated 3σ with time.

**Figure 6:**
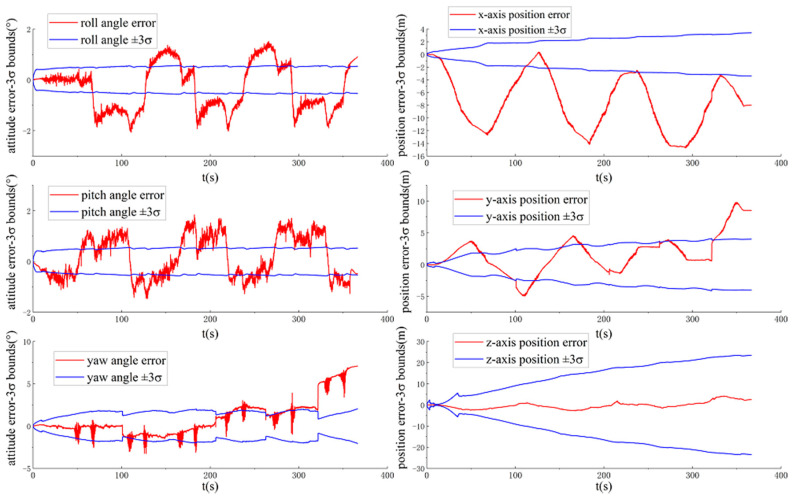
The ground-truth pose errors and estimated ±3σ bounds of ACK-MSCKF(3) with time for the VD06 dataset.

**Figure 7:**
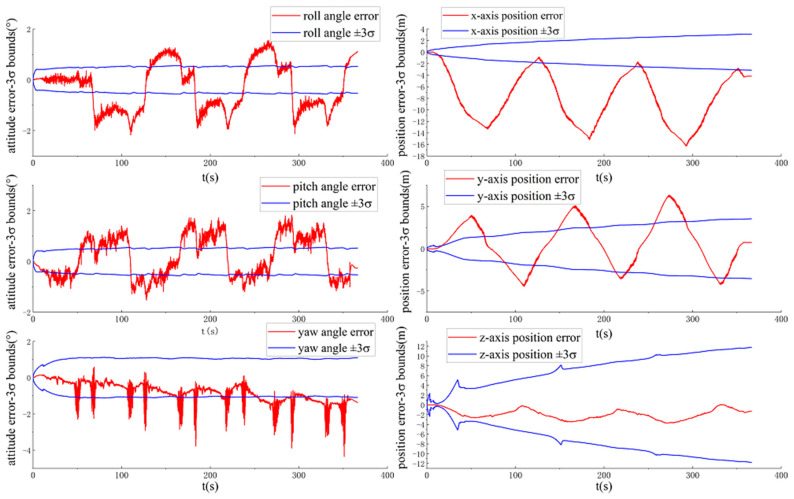
The ground-truth pose errors and estimated ±3σ bounds of ACK-MSCKF(4) with time for the VD06 dataset.

Figure 6 and Figure 7 show the ground-truth pose errors and estimated ±3σ bounds of ACK-MSCKF(3) and ACK-MSCKF(4) with time, respectively.

Similarly, it is seen from Figure 6 and Figure 7 that ACK-MSCKF(3) and ACK-MSCKF(4) are subjected to the same problem as above, especially along the direction of the vehicle yaw angle. From the above analysis, there appears the problem of estimator inconsistency in all the tunable parameter configurations of ACK-MSCKF.

**Figure 8:**
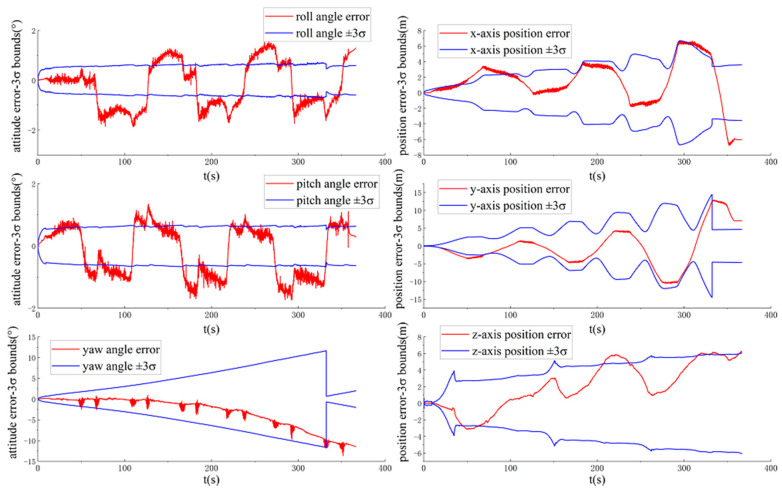
The ground-truth pose errors and estimated ±3σ bounds of MAVIO(1) with time for the VD06 dataset.

**Figure 9:**
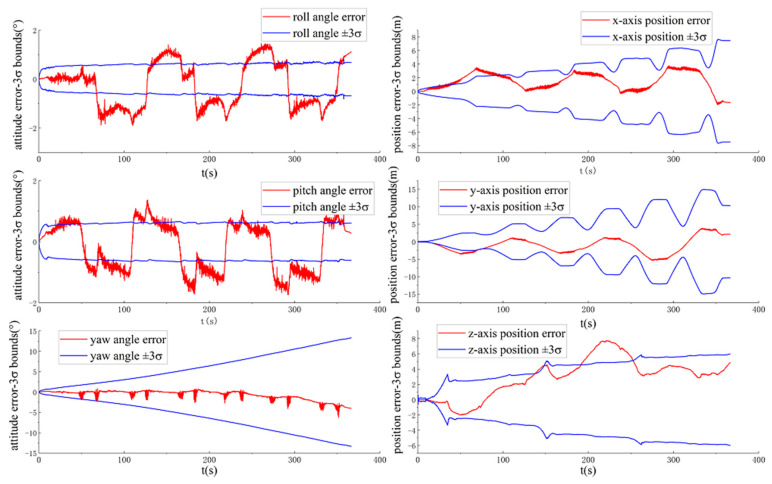
The ground-truth pose errors and estimated ±3σ bounds of MAVIO(2) with time for the VD06 dataset.

Figure 8 and Figure 9 show the ground-truth pose errors and estimated ±3σ bounds of MAVIO(1) and MAVIO(2) with time, respectively.

It is concluded from Figure 8 and Figure 9 that the estimator of MAVIO(1) and MAVIO(2) is consistent according to the fact that the ground-truth errors of the vehicle yaw angle and three-axis position are enclosed in their estimated ±3σ with time. Moreover, the three-axis vehicle position error state and yaw angle error state of the different parameter configurations of MAVIO are unobservable, which agrees with the result of the existing VIO observability analysis [64]. Therefore, MAVIO can overcome the inconsistency problem of ACK-MSCKF by introducing the vehicle velocity and angular rate error measurements, which could have a positive effect on improving positioning accuracy. Note that the reset phenomenon of the estimated ±3σ is attributed to the robust implementation of S-MSCKF [12].

**Figure 10:**
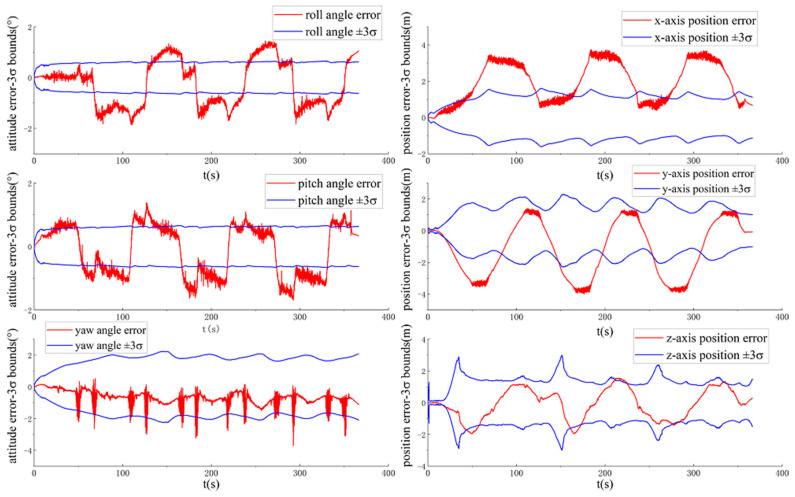
The ground-truth pose errors and estimated ±3σ bounds of the MAVIO-Global Navigation Satellite System (GNSS) with time for the VD06 dataset.

Figure 10 shows the ground-truth pose errors and estimated ±3σ bounds of MAVIO-GNSS with time.

It can be seen from Figure 10 that the three-axis vehicle position error state and yaw angle error state of MAVIO-GNSS are observable, which benefits from the raw GNSS error measurement model. This implies that MAVIO-GNSS can obtain a global position for a vehicle under a long-distance driving state.

By averaging all rounds of experimental results, the better performance of the RMSSR among the different configurations at the last time step for each experimental dataset is shown in Table 4.

It can be seen from the quantitative results in Table 4 that the average RMSSRs of MAVIO and ACK-MSCKF are smaller than the RMSSR for S-MSCKF for all of the datasets. Taken together, the above results indicate that both MAVIO and ACK-MSCKF can improve the observability of the VIO scale direction.

#### 3.2.2. Positioning Accuracy Comparison

**Figure 11:**
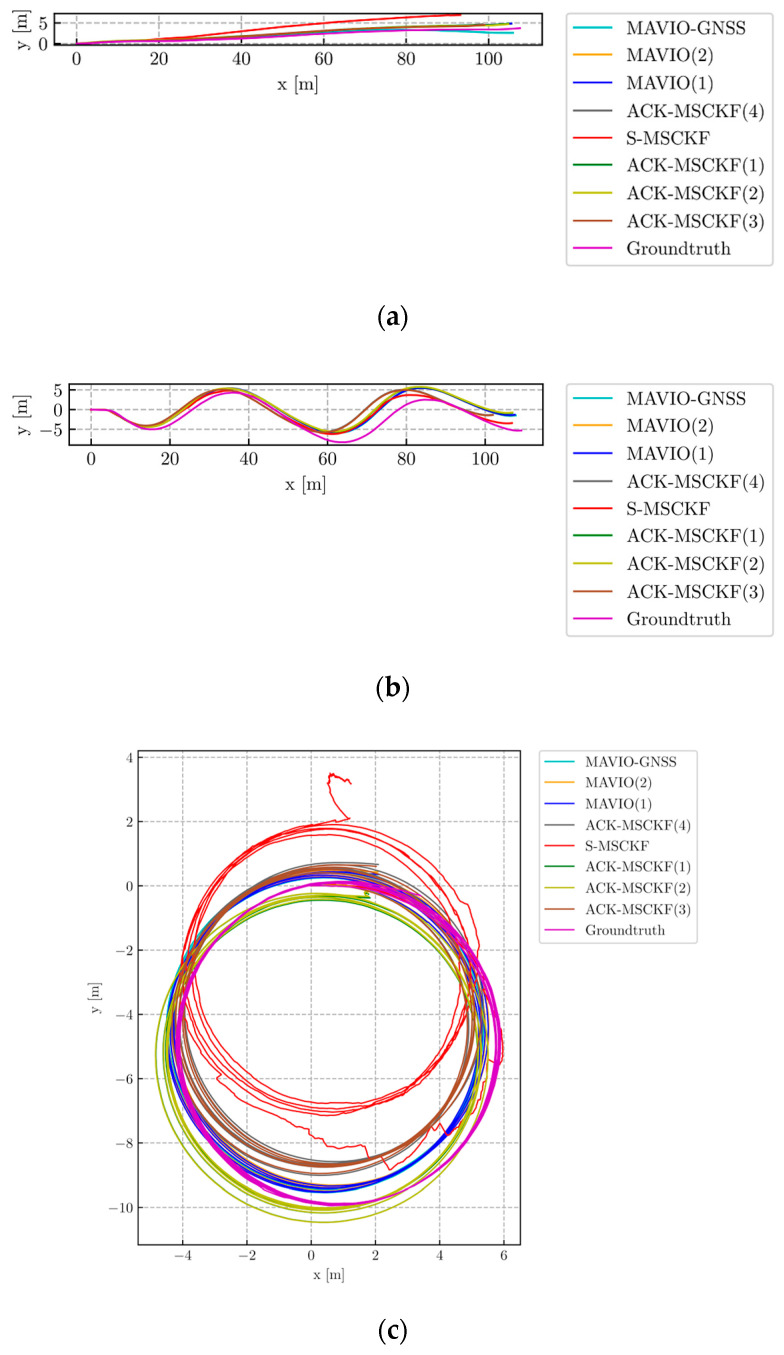

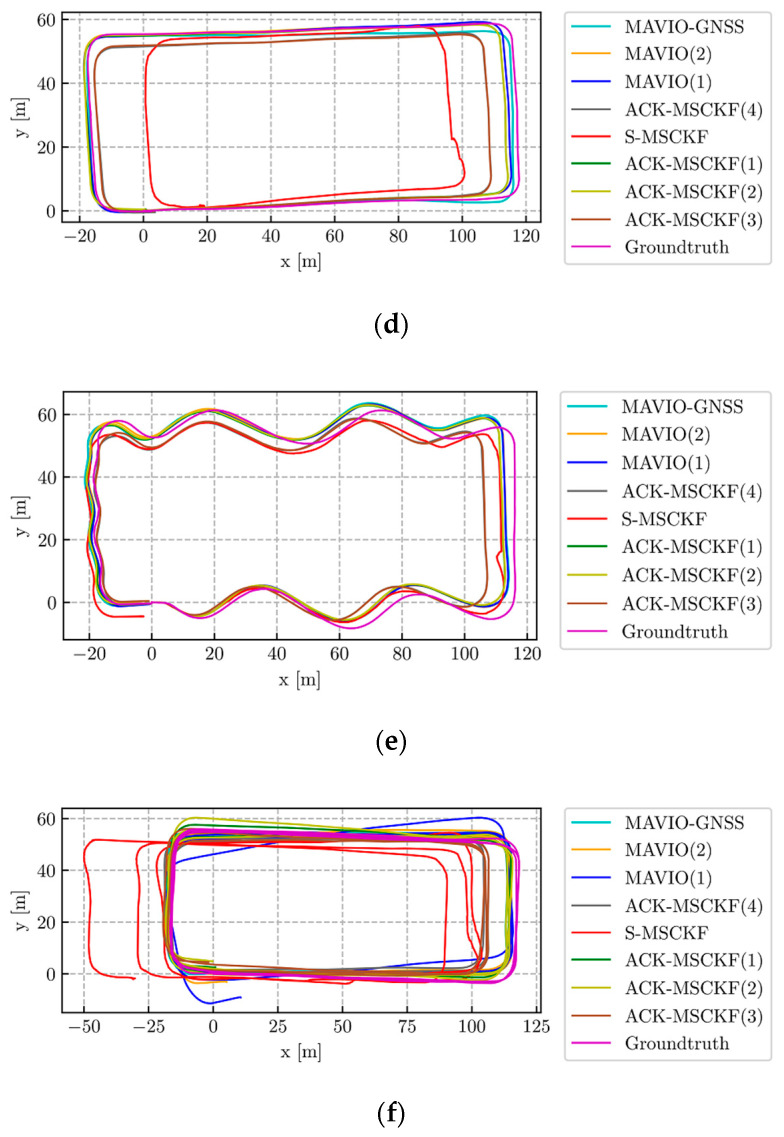
The top-views of estimated trajectories from MAVIO, MAVIO-GNSS, ACK-MSCKF, S-MSCKF and Groundtruth for the datasets: (**a**) VD01; (**b**) VD02; (**c**) VD03; (**d**) VD04; (**e**) VD05; (**f**) VD06.

Figure 11 shows the vehicle trajectory estimation results with a top view.

Figure 11 shows that S-MSCKF has larger scale drift, especially for the VD01, VD03, VD04 and VD06 datasets. Meanwhile, by selecting the appropriate parameter configuration in practice, the estimated trajectories of ACK-MSCKF and proposed MAVIO align to the ground truth better than S-MSCKF for all of the datasets. The performance difference among the different parameter configurations of ACK-MSCKF could be attributed to the inconsistency problem mentioned above. By improving the consistency, MAVIO is more robust than ACK-MSCKF. By introducing the raw GNSS error measurements, MAVIO-GNSS performs better than MAVIO, especially for the long-distance VD06 dataset.

**Figure 12:**
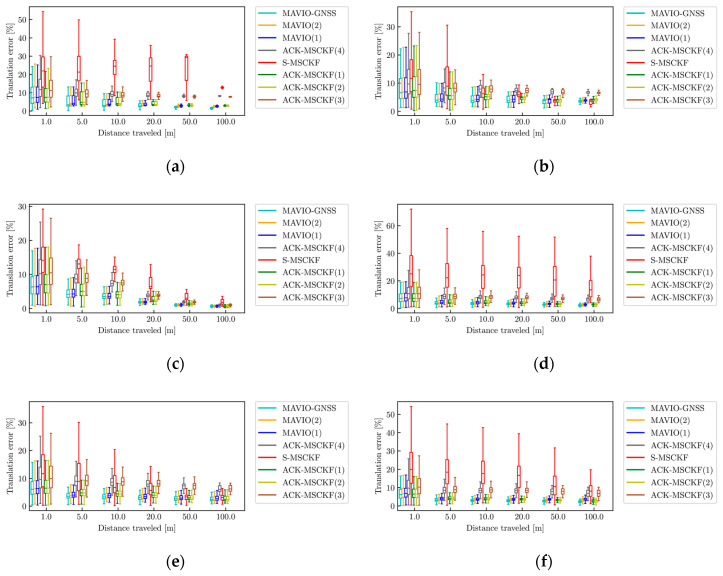
The relative translation error percentages (RTEPs) for MAVIO, MAVIO-GNSS, ACK-MSCKF and S-MSCKF for the datasets: (**a**) VD01; (**b**) VD02; (**c**) VD03; (**d**) VD04; (**e**) VD05; (**f**) VD06.

The boxplots of the RTEPs are shown in Figure 12.

Figure 12 demonstrates that the RTEPs of ACK-MSCKF and the proposed MAVIO are significantly reduced in comparison to the RTEP of S-MSCKF within the sub-trajectory length of 5 m for all of the datasets. Based on the average results for twenty rounds of experiments, the better performance of ATE among the different configurations along the whole trajectory for each experimental dataset is shown in Table 5, and Table 6 shows the overall RTE for all of the experimental datasets.

The quantitative results in Table 5 show that the average ATEs of MAVIO, MAVIO-GNSS and ACK-MSCKF is smaller than the ATE of S-MSCKF for all of the datasets but VD02. The trajectory error difference of S-MSCKF with that in [48] for the VD05 dataset is attributed to the data average processing which ensures more generalizable result. Compared with S-MSCKF, MAVIO reduces the average ATE by 86.61%, 68.03%, 86.45%, 25.65% and 78.26% for the VD01, VD03, VD04, VD05 and VD06 datasets, respectively. Moreover, MAVIO performs better than ACK-MSCKF for the VD01, VD02, VD03, VD04 and VD05 datasets, which reduces the average ATEs by 18.13%, 4.67%, 10.34%, 18.29% and 13.90%, respectively. It is also observable that MAVIO performs worse than ACK-MSCKF for the VD06 dataset. A potential reason is that the extrinsic parameters between {B} and {I} are not accurate enough. Furthermore, MAVIO-GNSS performs better than MAVIO for the VD01, VD02, VD04 and VD06 datasets, which reduces the average ATEs by 6.87%, 7.35%, 16.92% and 30.66%, respectively.

Table 6 shows that the overall RTE of MAVIO within the sub-trajectory length of 20 m is smaller than that of ACK-MSCKF. MAVIO-GNSS performs best in terms of the RTE within the sub-trajectory length of 100 m. The lowest RTE from MAVIO-GNSS is 2.29 m in the sub-trajectory length of 100 m, which is reduced, respectively, by 17.92%, 10.89% and 72.51% under the same conditions compared to that with MAVIO, ACK-MSCKF and S-MSCKF.

## 4. Discussion

ACK-MSCKF significantly improves the observability of the VIO scale direction and positioning accuracy under degenerate motions of ground vehicles. However, for practical implementation, the appropriate parameter configuration is needed to obtain better performance due to the inconsistency problem.

By improving the observability of the VIO scale direction and overcoming the inconsistency of ACK-MSCKF, MAVIO is more robust and further improves vehicle positioning accuracy using only a monocular configuration, which mainly benefits from the vehicle velocity and yaw angular rate error measurement model instead of the relative kinematic error measurement model for the vehicle. Compared with S-MSCKF, MAVIO reduces the average ATE by 86.61%, 68.03%, 86.45%, 25.65% and 78.26% for the VD01, VD03, VD04, VD05 and VD06 datasets, respectively. Moreover, MAVIO performs better than ACK-MSCKF for the VD01, VD02, VD03, VD04 and VD05 datasets, which reduces the average ATE by 18.13%, 4.67%, 10.34%, 18.29% and 13.90%, respectively. By introducing the raw GNSS error measurement model, MAVIO-GNSS further improves the vehicle positioning accuracy under a long-distance driving state. The lowest RTE from MAVIO-GNSS is 2.29 m in the sub-trajectory length of 100 m, and it is reduced, respectively, by 17.92%, 10.89% and 72.51% under the same conditions compared to the values obtained with MAVIO, ACK-MSCKF and S-MSCKF.

## 5. Conclusions

This paper proposed a consistent monocular Ackermann VIO termed MAVIO. The vehicle velocity and yaw angular rate error measurement model was introduced in detail, considering the lever arm effect between {B} and {I}. To obtain a global position for the vehicle, the raw GNSS error measurement model was introduced to further improve the performance of MAVIO. The observability and positioning accuracy were comprehensively compared using real-world datasets, averaging twenty rounds of experimental results. The experimental results demonstrated that the proposed MAVIO not only improved the observability of the VIO scale direction under degenerate motions of ground vehicles, but also resolved the inconsistency problem of ACK-MSCKF to further improve the vehicle positioning accuracy. Moreover, MAVIO-GNSS further improved the vehicle positioning accuracy under a long-distance driving state.

A limitation derives from the assumptions of approximate planar motion for a low-speed driving state and highly accurate extrinsic parameters between {B} and {I}. A future research target is to further investigate the performance impact of the vehicle speed and vehicle-IMU extrinsic parameter calibration to better enhance the positioning accuracy.

## Figures and Tables

**Table 1:** The tunable parameter configurations of ACK-MSCKF.

ACK-MSCKF	Tunable Parameter Configurations
ACK-MSCKF(1)	For Equations (A24) and (A31), $H_{\left(1,5\right)}^{B_{j,\theta }}=H_{\left(2,5\right)}^{B_{j,v}}=H_{\left(2,6\right)}^{B_{j,v}}=0_{3\times 3}$
ACK-MSCKF(2)	For Equations (A24) and (A31), $H_{\left(1,2\right)}^{B_{j,\theta }}=H_{\left(1,5\right)}^{B_{j,\theta }}=H_{\left(1,7\right)}^{B_{j,\theta }}=H_{\left(2,5\right)}^{B_{j,v}}=H_{\left(2,6\right)}^{B_{j,v}}=0_{3\times 3}$
ACK-MSCKF(3)	Same as Equations (A24) and (A31)
ACK-MSCKF(4)	For Equation (A24), $H_{\left(1,5\right)}^{B_{j,\theta }}=0_{3\times 3}$

**Table 2:** The tunable parameter configurations of the monocular Ackermann visual–inertial odometry (VIO) (MAVIO).

MAVIO	Tunable Parameter Configurations
MAVIO(1)	Same as Equations (A35) and (A40)
MAVIO(2)	For Equation (A35), $H_{\left(1,4\right)}^{B_{k},\omega }=H_{\left(1,6\right)}^{B_{k},\omega }=0_{3\times 3}$

**Table 3:** List of the real-world experimental datasets adapted from [54].

Dataset	Vehicle Driving State	Travel Duration (s)	Travel Distance (m)	Data Bulk			
				Vehicle CAN-Bus	Stereo Images	IMU	Ground Truth
VD01	Straight	54	109	8657	1615	10,808	10,818
VD02	S-shaped	60	122	9644	1820	12,146	12,186
VD03	Circular	99	162	15,631	2959	19,716	19,789
VD04	Straight and Turning	151	371	24,171	4532	30,164	30,244
VD05	S-shaped and Straight and Turning	170	400	27,135	5102	33,933	34,025
VD06	Straight and Turning	367	1085	58,463	11,014	73,169	73,386

**Table 4:** The average root mean square scale ratio (RMSSR) at the last time step on each experimental dataset.

Methods	VD01	VD02	VD03	VD04	VD05	VD06
MAVIO	1.29 ^(a)^	1.29 ^(a,b)^	1.14 ^(b)^	1.42 ^(a)^	1.42 ^(b)^	1.63 ^(a)^
ACK-MSCKF	1.21 ^(f)^	1.11 ^(f)^	0.98 ^(d,e,f)^	1.31 ^(f)^	1.21 ^(f)^	1.46 ^(f)^
S-MSCKF	1.68	1.38	1.22	1.99	1.57	2.08

^(a)^ From MAVIO(1); ^(b)^ From MAVIO(2); ^(c)^ From ACK-MSCKF(1); ^(d)^ From ACK-MSCKF(2); ^(e)^ From ACK-MSCKF(3); ^(f)^ From ACK-MSCKF(4).

**Table 5:** The average absolute trajectory error (ATE) along the whole trajectory for each experimental dataset.

Methods	VD01 (m)	VD02 (m)	VD03 (m)	VD04 (m)	VD05 (m)	VD06 (m)
MAVIO	1.31 ^(b)^	2.45 ^(b)^	0.78 ^(a)^	2.01 ^(b)^	3.16 ^(b)^	4.73 ^(b)^
MAVIO-GNSS	1.22	2.27	0.79	1.67	3.59	3.28
ACK-MSCKF	1.60 ^(d)^	2.57 ^(c)^	0.87 ^(d)^	2.46 ^(c,d)^	3.67 ^(d)^	3.80 ^(d)^
S-MSCKF	9.78	2.31	2.44	14.83	4.25	21.76

^(a)^ From MAVIO(1); ^(b)^ From MAVIO(2); ^(c)^ From ACK-MSCKF(1); ^(d)^ From ACK-MSCKF(2); ^(e)^ From ACK-MSCKF(3); ^(f)^ From ACK-MSCKF(4).

**Table 6:** The overall relative translation error (RTE) for all of the experimental datasets.

Sub-Trajectory Length (m)		Relative Translation Error (m)		
	MAVIO	MAVIO-GNSS	ACK-MSCKF	S-MSCKF
1	0.08 ^(a, b)^	0.08	0.08 ^(c, d)^	0.22
5	0.23 ^(b)^	0.21	0.26 ^(c, d)^	0.93
10	0.40 ^(b)^	0.35	0.44 ^(c, d)^	1.71
20	0.70 ^(b)^	0.60	0.76 ^(d)^	3.00
50	1.51 ^(b)^	1.29	1.51 ^(d)^	5.57
100	2.79 ^(b)^	2.29	2.57 ^(d)^	8.33

^(a)^ From MAVIO(1); ^(b)^ From MAVIO(2); ^(c)^ From ACK-MSCKF(1); ^(d)^ From ACK-MSCKF(2); ^(e)^ From ACK-MSCKF(3); ^(f)^ From ACK-MSCKF(4).

## Appendix A

According to the derivation of Equation (177) in [53], θ˜˙GBBk is derived as

(A1) θ˜˙GBBk=−⌊ω^Bk×⌋θ˜GBBk+ω˜Bk

where ω˜Bk is derived from the following formula:

(A2) ωBk=C(qIBk)ωIk

According to the error quaternion representation [53], Equation (A2) is represented as

(A3) ω^Bk+ω˜Bk=(I3−⌊θ˜IBk×⌋)C(q^IBk)(ω^Ik+ω˜Ik)

Then,

(A4) ω˜Bk=C(q^IBk)ω˜Ik−⌊θ˜IBk×⌋C(q^IBk)ω^Ik−⌊θ˜IBk×⌋C(q^IBk)ω˜Ik

By ignoring the second order terms, Equation (A4) is represented as

(A5) ω˜Bk=C(q^IBk)ω˜Ik−⌊θ˜IBk×⌋C(q^IBk)ω^Ik=⌊C(q^IBk)ω^Ik×⌋θ˜IBk+C(q^IBk)(−b˜g,k−ng,k)

Thus, Equation (A1) is represented as

(A6) θ˜˙GBBk=−⌊ω^Bk×⌋θ˜GBBk+⌊C(q^IBk)ω^Ik×⌋θ˜IBk+C(q^IBk)(−b˜g,k−ng,k)

Therefore, the Jacobian matrix of θ˜˙GBBk is represented as

(A7) {F(1,1)Bk=∂θ˜˙GBBk∂θ˜GBBk=−⌊C(q^IBk)ω^Ik×⌋=−⌊ω^Bk×⌋F(1,4)Bk=∂θ˜˙GBBk∂θ˜IBk=⌊C(q^IBk)ω^Ik×⌋=⌊ω^Bk×⌋F(1,6)Bk=∂θ˜˙GBBk∂b˜g,k=−C(q^IBk)G(1,1)Bk=∂θ˜˙GBBk∂ng,k=−C(q^IBk)

Following Equation (6), p˙GBBk is calculated as

(A8) p˙GBBk=vGBIk+C(qGBBk)TC(qIBk)(⌊ωIk×⌋pIB,k)

Equation (A8) is represented as

(A9) p^˙GBBk+p˜˙GBBk=v^GBIk+v˜GBIk+C(q^GBBk)T(I3+⌊θ˜GBBk×⌋)(I3−⌊θ˜IBk×⌋)C(q^IBk)⌊ω^Ik+ω˜Ik×⌋(p^IB,k+p˜IB,k)=v^GBIk+v˜GBIk+(C(q^GBBk)TC(q^IBk)−C(q^GBBk)T⌊θ˜IBk×⌋C(q^IBk)+C(q^GBBk)T⌊θ˜GBBk×⌋C(q^IBk))⋅(⌊ω^Ik×⌋p^IB,k+⌊ω^Ik×⌋p˜IB,k+⌊ω˜Ik×⌋p^IB,k)=v^GBIk+v˜GBIk+C(q^GBBk)TC(q^IBk)⌊ω^Ik×⌋p^IB,k+C(q^GBBk)TC(q^IBk)⌊ω^Ik×⌋p˜IB,k+C(q^GBBk)TC(q^IBk)⌊ω˜Ik×⌋p^IB,k−C(q^GBBk)T⌊θ˜IBk×⌋C(q^IBk)⌊ω^Ik×⌋p^IB,k+C(q^GBBk)T⌊θ˜GBBk×⌋C(q^IBk)⌊ω^Ik×⌋p^IB,k

Then,

(A10) p˜˙GBBk=−C(q^GBBk)T⌊C(q^IBk)⌊ω^Ik×⌋p^IB,k×⌋θ˜GBBk+v˜GBIk+C(q^GBBk)T⌊C(q^IBk)⌊ω^Ik×⌋p^IB,k×⌋θ˜IBk+C(q^GBBk)TC(q^IBk)⌊ω^Ik×⌋p˜IB,k−C(q^GBBk)TC(q^IBk)⌊p^IB,k×⌋(−b˜g,k−ng,k)

Therefore, the Jacobian matrix of p˜˙GBBk is represented as

(A11) {F(2,1)Bk=∂p˜˙GBBk∂θ˜GBBk=−C(q^GBBk)T⌊(C(q^IBk)⌊ω^Ik×⌋p^IB,k)×⌋F(2,3)Bk=∂p˜˙GBBk∂v˜GBIk=I3F(2,4)Bk=∂p˜˙GBBk∂θ˜IBk=C(q^GBBk)T⌊(C(q^IBk)⌊ω^Ik×⌋p^IB,k)×⌋F(2,5)Bk=∂p˜˙GBBk∂p˜IB,k=C(q^GBBk)TC(q^IBk)⌊ω^Ik×⌋F(2,6)Bk=∂p˜˙GBBk∂b˜g,k=C(q^GBBk)TC(q^IBk)⌊p^IB,k×⌋G(2,1)Bk=∂p˜˙GBBk∂ng,k=C(q^GBBk)TC(q^IBk)⌊p^IB,k×⌋

Following Equation (6), v˙GBIk is calculated as

(A12) v˙GBIk=C(qGBBk)TC(qIBk)aIk+gG

Equation (A12) is represented as

(A13) v^˙GBIk+v˜˙GBIk=C(q^GBBk)T(I3+⌊θ˜GBBk×⌋)(I3−⌊θ˜IBk×⌋)C(q^IBk)(a^Ik+a˜Ik)+gG=(C(q^GBBk)T−C(q^GBBk)T⌊θ˜IBk×⌋+C(q^GBBk)T⌊θ˜GBBk×⌋)(C(q^IBk)a^Ik+C(q^IBk)a˜Ik)+gG=C(q^GBBk)TC(q^IBk)a^Ik+C(q^GBBk)TC(q^IBk)a˜Ik−C(q^GBBk)T⌊θ˜IBk×⌋C(q^IBk)a^Ik+C(q^GBBk)T⌊θ˜GBBk×⌋C(q^IBk)a^Ik+gG

Then,

(A14) v˜˙GBIk=−C(q^GBBk)T⌊C(q^IBk)a^Ik×⌋θ˜GBBk+C(q^GBBk)T⌊C(q^IBk)a^Ik×⌋θ˜IBk+C(q^GBBk)TC(q^IBk)a˜Ik=−C(q^GBBk)T⌊C(q^IBk)a^Ik×⌋θ˜GBBk+C(q^GBBk)T⌊C(q^IBk)a^Ik×⌋θ˜IBk+C(q^GBBk)TC(q^IBk)(−b˜a,k−na,k)

Therefore, the Jacobian matrix of v˜˙GBIk is represented as

(A15) {F(3,1)Bk=∂v˜˙GBIk∂θ˜GBBk=−C(q^GBBk)T⌊C(q^IBk)a^Ik×⌋F(3,4)Bk=∂v˜˙GBIk∂θ˜IBk=C(q^GBBk)T⌊C(q^IBk)a^Ik×⌋F(3,7)Bk=∂v˜˙GBIk∂b˜a,k=−C(q^GBBk)TC(q^IBk)G(3,3)Bk=∂v˜˙GBIk∂na,k=−C(q^GBBk)TC(q^IBk)

According to the characteristics of the IMU noise model [53],

(A16) $$\begin{array}{l} {\dot{b}}_{g,k}^{}=n_{\omega g,k}^{},\;{\dot{b}}_{a,k}^{}=n_{\omega a,k}^{}\\ {\dot{\hat{b}}}_{g,k}^{}=0_{3\times 1},\;{\dot{\hat{b}}}_{a,k}^{}=0_{3\times 1} \end{array}$$

Equation (A16) can be represented as

(A17) $$\begin{array}{l} {\dot{\tilde{b}}}_{g,k}^{}=n_{\omega g,k}^{}\\ {\dot{\tilde{b}}}_{a,k}^{}=n_{\omega g,k}^{} \end{array}$$

Therefore,

(A18) $$\left\{ \begin{array}{l} G_{\left(6,2\right)}^{B_{k}}=\frac{\partial {\dot{\tilde{b}}}_{g,k}^{}}{\partial n_{\omega g,k}^{}}=I_{3}\\ G_{\left(7,4\right)}^{B_{k}}=\frac{\partial {\dot{\tilde{b}}}_{a,k}^{}}{\partial n_{\omega a,k}^{}}=I_{3} \end{array}\right.$$

Thereby, the analytical expressions of each block matrix in Equations (9) and (10) are derived.

## Appendix B

Following Equation (7) in [48], according to the error quaternion representation [53], the Ackermann steering attitude angle error θ˜BjBj−1 from time $t_{j-1}$ to time $t_{j}$ is derived as

(A19) (I3×3−⌊θ˜BjBj−1×⌋)C(q^BjBj−1)=C(q^BC)T(I3×3+⌊θ˜IC×⌋)(I3×3−⌊θ˜GCj−1×⌋)C(q^GCj−1)C(q^GCj)T(I3×3+⌊θ˜GCj×⌋)(I3×3−⌊θ˜IC×⌋)C(q^BC)=(C(q^BC)TC(q^GCj−1)C(q^GCj)T−C(q^BC)T⌊θ˜GCj−1×⌋C(q^GCj−1)C(q^GCj)T+C(q^BC)T⌊θ˜IC×⌋C(q^GCj−1)C(q^GCj)T)(C(q^BC)−⌊θ˜IC×⌋C(q^BC)+⌊θ˜GCj×⌋C(q^BC))=(C(q^BC)TC(q^CjCj−1)−C(q^BC)T⌊θ˜GCj−1×⌋C(q^CjCj−1)+C(q^BC)T⌊θ˜IC×⌋C(q^CjCj−1))(C(q^BC)−⌊θ˜IC×⌋C(q^BC)+⌊θ˜GCj×⌋C(q^BC))=C(q^BC)TC(q^CjCj−1)C(q^BC)−C(q^BC)TC(q^CjCj−1)⌊θ˜IC×⌋C(q^BC)+C(q^BC)TC(q^CjCj−1)⌊θ˜GCj×⌋C(q^BC)−C(q^BC)T⌊θ˜GCj−1×⌋C(q^CjCj−1)C(q^BC)+C(q^BC)T⌊θ˜IC×⌋C(q^CjCj−1)C(q^BC)

Equation (A19) can be represented as

(A20) ⌊θ˜BjBj−1×⌋C(q^BjBj−1)=C(q^BC)TC(q^CjCj−1)⌊θ˜IC×⌋C(q^BC)−C(q^BC)TC(q^CjCj−1)⌊θ˜GCj×⌋C(q^BC)+C(q^BC)T⌊θ˜GCj−1×⌋C(q^CjCj−1)C(q^BC)−C(q^BC)T⌊θ˜IC×⌋C(q^CjCj−1)C(q^BC)

Then,

(A21) ⌊θ˜BjBj−1×⌋=C(q^BC)TC(q^CjCj−1)⌊θ˜IC×⌋C(q^CjCj−1)TC(q^BC)−C(q^BC)TC(q^CjCj−1)⌊θ˜GCj×⌋C(q^CjCj−1)TC(q^BC)+C(q^BC)T⌊θ˜GCj−1×⌋C(q^BC)−C(q^BC)T⌊θ˜IC×⌋C(q^BC)

According to the properties of the cross product skew-symmetric matrix [53], Equation (A21) is represented as

(A22) ⌊θ˜BjBj−1×⌋=⌊C(q^BC)TC(q^CjCj−1)θ˜IC×⌋−⌊C(q^BC)TC(q^CjCj−1)θ˜GCj×⌋+⌊C(q^BC)Tθ˜GCj−1×⌋−⌊C(q^BC)Tθ˜IC×⌋

Then,

(A23) θ˜BjBj−1=−C(q^BC)TC(q^CjCj−1)θ˜GCj+C(q^BC)Tθ˜GCj−1+(C(q^BC)TC(q^CjCj−1)−C(q^BC)T)θ˜IC

Therefore, the measurement Jacobian matrixes of the vehicle relative rotation errors are represented as

(A24) {H(1,7)Bj,θ=∂rθj−1,jB∂θ˜GCj=∂θ˜BjBj−1∂θ˜GCj=−C(q^BC)TC(q^CjCj−1)=−C(q^BC)TC(q^GCj−1)C(q^GCj)TH(1,2)Bj,θ=∂rθj−1,jB∂θ˜IC=∂θ˜BjBj−1∂θ˜IC=C(q^BC)TC(q^CjCj−1)−C(q^BC)T=C(q^BC)TC(q^GCj−1)C(q^GCj)T−C(q^BC)TH(1,5)Bj,θ=∂rθj−1,jB∂θ˜GCj−1=∂θ˜BjBj−1∂θ˜GCj−1=C(q^BC)T

Similarly, the velocity measurement residual $r_{v_{j}}^{B}$ at time $t_{j}$ is represented as

(A25) rvjB=zvjB−p^Bj−1Bj/Δtj−1,j

Then,

(A26) rvjBΔtj−1,j=zvjBΔtj−1,j−p^Bj−1Bj=pBj−1Bj−p^Bj−1Bj

Equation (A26) is represented as

(A27) rvjBΔtj−1,j=pBj−1Bj−p^Bj−1Bj=C(qBC)T[C(qGCj−1)[C(qGCj)TpCB+pGCj−pGCj−1]−pCB]−p^Bj−1Bj

where

(A28) pCB=pCI+C(qIC)pIB=−C(qIC)pIC+C(qIC)pIB=C(qIC)(−pIC+pIB)

Thus, Equation (A27) is represented as

(A29) rvjBΔtj−1,j=C(qBC)T[C(qGCj−1)[C(qGCj)TpCB+pGCj−pGCj−1]−pCB]−p^Bj−1Bj=C(q^BC)T(I3×3+⌊θ˜IC×⌋)(I3×3−⌊θ˜GCj−1×⌋)C(q^GCj−1)⋅[C(q^GCj)T(I3×3+⌊θ˜GCj×⌋)(I3×3−⌊θ˜IC×⌋)C(q^IC)(−p^IC−p˜IC+pIB)+p^GCj+p˜GCj−p^GCj−1−p˜GCj−1]−C(q^BC)T(I3×3+⌊θ˜IC×⌋)(I3×3−⌊θ˜IC×⌋)C(q^IC)(−p^IC−p˜IC+pIB)−p^Bj−1Bj=C(q^BC)T(I3×3−⌊θ˜GCj−1×⌋+⌊θ˜IC×⌋)C(q^GCj−1)⋅[(C(q^GCj)TC(q^IC)−C(q^GCj)T⌊θ˜IC×⌋C(q^IC)+C(q^GCj)T⌊θ˜GCj×⌋C(q^IC))(−p^IC−p˜IC+pIB)+p^GCj+p˜GCj−p^GCj−1−p˜GCj−1]−(C(q^BC)TC(q^IC)−C(q^BC)T⌊θ˜IC×⌋C(q^IC)+C(q^BC)T⌊θ˜IC×⌋C(q^IC))(−p^IC−p˜IC+pIB)−p^Bj−1Bj=(C(q^BC)TC(q^GCj−1)−C(q^BC)T⌊θ˜GCj−1×⌋C(q^GCj−1)+C(q^BC)T⌊θ˜IC×⌋C(q^GCj−1))⋅[(C(q^GCj)TC(q^IC)−C(q^GCj)T⌊θ˜IC×⌋C(q^IC)+C(q^GCj)T⌊θ˜GCj×⌋C(q^IC))(−p^IC−p˜IC+pIB)+p^GCj+p˜GCj−p^GCj−1−p˜GCj−1]−(C(q^BC)TC(q^IC)(−p^IC+pIB)−C(q^BC)TC(q^IC)p˜IC)−p^Bj−1Bj=(C(q^BC)TC(q^GCj−1)−C(q^BC)T⌊θ˜GCj−1×⌋C(q^GCj−1)+C(q^BC)T⌊θ˜IC×⌋C(q^GCj−1))⋅(C(q^GCj)Tp^CB−C(q^GCj)TC(q^IC)p˜IC−C(q^GCj)T⌊θ˜IC×⌋p^CB+C(q^GCj)T⌊θ˜GCj×⌋p^CB+p^GCj+p˜GCj−p^GCj−1−p˜GCj−1)−C(q^GCj)Tp^CB+C(q^BC)TC(q^IC)p˜IC−p^Bj−1Bj=C(q^BC)TC(q^GCj−1)C(q^GCj)Tp^CB−C(q^BC)TC(q^GCj−1)C(q^GCj)TC(q^IC)p˜IC−C(q^BC)TC(q^GCj−1)C(q^GCj)T⌊θ˜IC×⌋p^CB+C(q^BC)TC(q^GCj−1)C(q^GCj)T⌊θ˜GCj×⌋p^CB+C(q^BC)TC(q^GCj−1)p^GCj+C(q^BC)TC(q^GCj−1)p˜GCj−C(q^BC)TC(q^GCj−1)p^GCj−1−C(q^BC)TC(q^GCj−1)p˜GCj−1−C(q^BC)T⌊θ˜GCj−1×⌋C(q^GCj−1)C(q^GCj)Tp^CB−C(q^BC)T⌊θ˜GCj−1×⌋C(q^GCj−1)(p^GCj−p^GCj−1)+C(q^BC)T⌊θ˜IC×⌋C(q^GCj−1)C(q^GCj)Tp^CB+C(q^BC)T⌊θ˜IC×⌋C(q^GCj−1)(p^GCj−p^GCj−1)−C(q^GCj)Tp^CB+C(q^BC)TC(q^IC)p˜IC−C(q^BC)T[C(q^GCj−1)[C(q^GCj)Tp^CB+p^GCj−p^GCj−1]−p^CB]=−C(q^BC)TC(q^GCj−1)C(q^GCj)TC(q^IC)p˜IC−C(q^BC)TC(q^GCj−1)C(q^GCj)T⌊θ˜IC×⌋p^CB+C(q^BC)TC(q^GCj−1)C(q^GCj)T⌊θ˜GCj×⌋p^CB+C(q^BC)TC(q^GCj−1)p˜GCj−C(q^BC)TC(q^GCj−1)p˜GCj−1−C(q^BC)T⌊θ˜GCj−1×⌋C(q^GCj−1)C(q^GCj)Tp^CB−C(q^BC)T⌊θ˜GCj−1×⌋C(q^GCj−1)(p^GCj−p^GCj−1)+C(q^BC)T⌊θ˜IC×⌋C(q^GCj−1)C(q^GCj)Tp^CB+C(q^BC)T⌊θ˜IC×⌋C(q^GCj−1)(p^GCj−p^GCj−1)+C(q^BC)TC(q^IC)p˜IC

Then,

(A30) rvjBΔtj−1,j=−C(q^BC)TC(q^GCj−1)C(q^GCj)T⌊p^CB×⌋θ˜GCj+C(q^BC)TC(q^GCj−1)p˜GCj+C(q^BC)T[C(q^GCj−1)C(q^GCj)T⌊p^CB×⌋−⌊(C(q^GCj−1)C(q^GCj)Tp^CB+C(q^GCj−1)(p^GCj−p^GCj−1))×⌋]θ˜IC+C(q^BC)T(C(q^IC)−C(q^GCj−1)C(q^GCj)TC(q^IC))p˜IC−C(q^BC)TC(q^GCj−1)p˜GCj−1+C(q^BC)T⌊(C(q^GCj−1)C(q^GCj)Tp^CB+C(q^GCj−1)(p^GCj−p^GCj−1))×⌋θ˜GCj−1

Therefore, the measurement Jacobian matrixes of the vehicle relative translation errors are represented as

(A31) {H(2,7)Bj,v=∂rvjB∂θ˜GCj=−C(q^BC)TC(q^GCj−1)C(q^GCj)T⌊p^CB×⌋/Δtj−1,jH(2,8)Bj,v=∂rvjB∂p˜CjG=C(q^BC)TC(q^GCj−1)/Δtj−1,jH(2,2)Bj,v=∂rvjB∂θ˜IC=C(q^BC)T[C(q^GCj−1)C(q^GCj)T⌊p^CB×⌋−⌊(C(q^GCj−1)C(q^GCj)Tp^CB+C(q^GCj−1)(p^GCj−p^GCj−1))×⌋]/Δtj−1,jH(2,3)Bj,v=∂rvjB∂p˜IC=C(q^BC)T(C(q^IC)−C(q^GCj−1)C(q^GCj)TC(q^IC))/Δtj−1,jH(2,5)Bj,v=∂rvjB∂θ˜GCj−1=C(q^BC)T⌊(C(q^GCj−1)C(q^GCj)Tp^CB+C(q^GCj−1)(p^GCj−p^GCj−1))×⌋/Δtj−1,jH(2,6)Bj,v=∂rvjB∂p˜GCj−1=−C(q^BC)TC(q^GCj−1)/Δtj−1,j

Thereby, the analytical expressions of all the block matrixes in Equation (15) are derived.

## Appendix C

Following Equation (16), the vehicle angular rate measurement residual rBωk at time $t_{k}$ in {B} is represented as

(A32) rBωk=ωBk−ω^Bk

Based on the lever arm effect between {B} and {I}, Equation (A32) is represented as

(A33) rBωk=ωBk−ω^Bk=C(qIBk)⋅ωIk−ω^Bk

According to the error quaternion representation [53], Equation (A33) is represented as

(A34) rBωk=C(qIBk)⋅ωIk−ω^Bk=(I3×3−⌊θ˜IBk×⌋)C(q^IBk)(ω^Ik+ω˜Ik)−ω^Bk=C(q^IBk)ω^Ik+C(q^IBk)ω˜Ik−⌊θ˜IBk×⌋C(q^IBk)ω^Ik−C(q^IBk)⋅ω^Ik=C(q^IBk)ω˜Ik−⌊θ˜IBk×⌋C(q^IBk)ω^Ik=⌊C(q^IBk)ω^Ik×⌋θ˜IBk+C(q^IBk)(−b˜g,k−ng,k)=⌊C(q^IBk)ω^Ik×⌋θ˜IBk−C(q^IBk)b˜g,k−C(q^IBk)ng,k

Therefore, the measurement Jacobian matrix of the vehicle angular rate errors is represented as

(A35) {H(1,4)Bk,ω=∂rBωk∂θ˜IBk=⌊C(q^IBk)ω^Ik×⌋H(1,6)Bk,ω=∂rBωk∂b˜g,k=−C(q^IBk)

Similarly, following Equation (16), the vehicle velocity measurement residual rBvk at time $t_{k}$ in {B} is represented as

(A36) rBvk=vBBk−v^BBk

Based on the lever arm effect between {B} and {I}, Equation (A36) is represented as

(A37) rBvk=vBBk−v^BBk=C(qGBBk)vGBIk+C(qIBk)(⌊ωIk×⌋pIB,k)−v^BBk

According to the error quaternion representation [53], Equation (A37) is represented as

(A38) rBvk=C(qGBBk)vGBIk+C(qIBk)(⌊ωIk×⌋pIB,k)−v^BBk=(I3×3−⌊θ˜GBBk×⌋)C(q^GBBk)(v^GBIk+v˜GBIk)+(I3×3−⌊θ˜IBk×⌋)C(q^IBk)(⌊ω^Ik×⌋+⌊ω˜Ik×⌋)(p^IB,k+p˜IB,k)−v^BBk=C(q^GBBk)v^GBIk+C(q^GBBk)v˜GBIk−⌊θ˜GBBk×⌋C(q^GBBk)v^GBIk−⌊θ˜GBBk×⌋C(q^GBBk)v˜GBIk+(C(q^IBk)−⌊θ˜IBk×⌋C(q^IBk))(⌊ω^Ik×⌋p^IB,k+⌊ω^Ik×⌋p˜IB,k+⌊ω˜Ik×⌋p^IB,k+⌊ω˜Ik×⌋p˜IB,k)−v^BBk

By ignoring the second order terms, Equation (A38) is represented as

(A39) rBvk=C(q^GBBk)v^GBIk+C(q^GBBk)v˜GBIk−⌊θ˜GBBk×⌋C(q^GBBk)v^GBIk+(C(q^IBk)−⌊θ˜IBk×⌋C(q^IBk))(⌊ω^Ik×⌋p^IB,k+⌊ω^Ik×⌋p˜IB,k+⌊ω˜Ik×⌋p^IB,k)−v^BBk=C(q^GBBk)v^GBIk+C(q^GBBk)v˜GBIk+⌊C(q^GBBk)v^GBIk×⌋θ˜GBBk+C(q^IBk)⌊ω^Ik×⌋p^IB,k++C(q^IBk)⌊ω^Ik×⌋p˜IB,k+C(q^IBk)⌊ω˜Ik×⌋p^IB,k−⌊θ˜IBk×⌋C(q^IBk)⌊ω^Ik×⌋p^IB,k−(C(q^GBBk)v^GBIk+C(q^IBk)(⌊ω^Ik×⌋p^IB,k))=C(q^GBBk)v˜GBIk+⌊C(q^GBBk)v^GBIk×⌋θ˜GBBk+C(q^IBk)⌊ω^Ik×⌋p˜IB,k+C(q^IBk)⌊ω˜Ik×⌋p^IB,k−⌊θ˜IBk×⌋C(q^IBk)⌊ω^Ik×⌋p^IB,k=C(q^GBBk)v˜GBIk+⌊C(q^GBBk)v^GBIk×⌋θ˜GBBk+⌊(C(q^IBk)⌊ω^Ik×⌋p^IB,k)×⌋θ˜IBk+C(q^IBk)⌊ω^Ik×⌋p˜IB,k−C(q^IBk)⌊p^IB,k×⌋ω˜Ik=C(q^GBBk)v˜GBIk+⌊C(q^GBBk)v^GBIk×⌋θ˜GBBk+⌊(C(q^IBk)⌊ω^Ik×⌋p^IB,k)×⌋θ˜IBk+C(q^IBk)⌊ω^Ik×⌋p˜IB,k−C(q^IBk)⌊p^IB,k×⌋(−b˜g,k−ng,k)=⌊C(q^GBBk)v^GBIk×⌋θ˜GBBk+C(q^GBBk)v˜GBIk+⌊(C(q^IBk)⌊ω^Ik×⌋p^IB,k)×⌋θ˜IBk+C(q^IBk)⌊ω^Ik×⌋p˜IB,k+C(q^IBk)⌊p^IB,k×⌋b˜g,k+C(q^IBk)⌊p^IB,k×⌋ng,k

Therefore, the measurement Jacobian matrix of the vehicle velocity errors is represented as

(A40) {H(2,1)Bk,v=∂rBvk∂θ˜GBBk=⌊C(q^GBBk)v^GBIk×⌋H(2,3)Bk,v=∂rBvk∂v˜GBIk=C(q^GBBk)H(2,4)Bk,v=∂rBvk∂θ˜IBk=⌊(C(q^IBk)⌊ω^Ik×⌋p^IB,k)×⌋H(2,5)Bk,v=∂rBvk∂p˜IB,k=C(q^IBk)⌊ω^Ik×⌋H(2,6)Bk,v=∂rBvk∂b˜g,k=C(q^IBk)⌊p^IB,k×⌋

Thereby, the analytical expressions of all the block matrixes in Equation (30) are derived.

## Appendix D

According to the error quaternion representation [53], Equation (31) is represented as

(A41) rUSpk=pUSSk−p^USSk=pUSGB+C(qUSGB)T(p^GBBk+p˜GBBk)+C(qUSGB)TC(q^GBBk)T(I3×3+⌊θ˜GBBk×⌋)(I3×3−⌊θ˜IBk×⌋)C(q^IBk)(pIS−p^IB,k−p˜IB,k)−p^USSk=pUSGB+C(qUSGB)Tp^GBBk+C(qUSGB)Tp˜GBBk+C(qUSGB)TC(q^GBBk)TC(q^IBk)pIS−C(qUSGB)TC(q^GBBk)TC(q^IBk)p^IB,k−C(qUSGB)TC(q^GBBk)TC(q^IBk)p˜IB,k−C(qUSGB)TC(q^GBBk)T⌊θ˜IBk×⌋C(q^IBk)pIS+C(qUSGB)TC(q^GBBk)T⌊θ˜IBk×⌋C(q^IBk)p^IB,k+C(qUSGB)TC(q^GBBk)T⌊θ˜GBBk×⌋C(q^IBk)pIS−C(qUSGB)TC(q^GBBk)T⌊θ˜GBBk×⌋C(q^IBk)p^IB,k−p^USSk

Following Equation (32), Equation (A41) is represented as

(A42) rUSpk=C(qUSGB)Tp˜GBBk−C(qUSGB)TC(q^GBBk)TC(q^IBk)p˜IB,k−C(qUSGB)TC(q^GBBk)T⌊θ˜IBk×⌋C(q^IBk)pIS+C(qUSGB)TC(q^GBBk)T⌊θ˜IBk×⌋C(q^IBk)p^IB,k+C(qUSGB)TC(q^GBBk)T⌊θ˜GBBk×⌋C(q^IBk)pIS−C(qUSGB)TC(q^GBBk)T⌊θ˜GBBk×⌋C(q^IBk)p^IB,k=−C(qUSGB)TC(q^GBBk)T⌊C(q^IBk)pIS×⌋θ˜GBBk+(qUSGB)TC(q^GBBk)T⌊C(q^IBk)p^IB,k×⌋θ˜GBBk+C(qUSGB)Tp˜GBBk+C(qUSGB)TC(q^GBBk)T⌊C(q^IBk)pIS×⌋θ˜IBk−C(qUSGB)TC(q^GBBk)T⌊C(q^IBk)p^IB,k×⌋θ˜IBk−−C(qUSGB)TC(q^GBBk)TC(q^IBk)p˜IB,k=C(qUSGB)TC(q^GBBk)T⌊C(q^IBk)(p^IB,k−pIS)×⌋θ˜GBBk+C(qUSGB)Tp˜GBBk+(qUSGB)TC(q^GBBk)T⌊C(q^IBk)(pIS−p^IB,k)×⌋θ˜IBk−C(qUSGB)TC(q^GBBk)TC(q^IBk)p˜IB,k

Therefore, the measurement Jacobian matrix of the raw GNSS errors is represented as

(A43) {H(1,1)Sk,p=∂rUSpk∂θ˜GBBk=C(qUSGB)TC(q^GBBk)T⌊C(q^IBk)(p^IB,k−pIS)×⌋H(1,2)Sk,p=∂rUSpk∂p˜GBBk=C(qUSGB)TH(1,4)Sk,p=∂rUSpk∂θ˜IBk=(qUSGB)TC(q^GBBk)T⌊C(q^IBk)(pIS−p^IB,k)×⌋H(1,5)Sk,p=∂rUSpk∂p˜IB,k=−C(qUSGB)TC(q^GBBk)TC(q^IBk)

Thereby, the analytical expressions of all the block matrixes in Equation (34) are derived.
